# Development of a Clinical Pathway and Technical Aspects of Upper Airway Stimulation Therapy for Obstructive Sleep Apnea

**DOI:** 10.3389/fnins.2017.00523

**Published:** 2017-09-21

**Authors:** Olivier M. Vanderveken, Jolien Beyers, Sara Op de Beeck, Marijke Dieltjens, Marc Willemen, Johan A. Verbraecken, Wilfried A. De Backer, Paul H. Van de Heyning

**Affiliations:** ^1^Faculty of Medicine and Health Sciences, University of Antwerp Antwerp, Belgium; ^2^Department ENT, Head and Neck Surgery, Antwerp University Hospital Antwerp, Belgium; ^3^Multidisciplinary Sleep Disorders Center, Antwerp University Hospital Antwerp, Belgium; ^4^Department of Pulmonary Medicine, Antwerp University Hospital Antwerp, Belgium

**Keywords:** care pathways, complete concentric collapse, drug-induced sleep endoscopy, hypoglossal nerve stimulation, neurostimulation, pathophysiology, sleep-disordered breathing

## Abstract

Obstructive sleep apnea (OSA) is a common disease with high morbidity and related mortality. Narrowing and collapse of the pharyngeal airway during sleep characterize the disease, resulting in a decrease (hypopnea) or a complete cessation (apnea) of oronasal airflow. Upper airway stimulation (UAS), using electrical neurostimulation of the hypoglossal nerve (n. XII) synchronized with ventilation, is a novel, evolving treatment option. UAS was found to be an effective treatment in CPAP-intolerant patients. The treatment success is partly due to the strict selection of the patients, based on previous findings. Furthermore, post-operative follow-up is needed in order to maintain or improve treatment outcome. Therefore, a clinical pathway, which provides structure and standardization, is crucial. In this paper, the aim is to discuss the technical aspects of UAS therapy and to describe a clinical pathway to organize the care process of UAS for OSA in a structured and standardized way.

## Introduction

The human upper airway (UA) contains a collapsible portion that extends from the hard palate to the larynx (Malhotra and White, 2002; Patil et al., 2007b; Eckert and Malhotra, 2008). The collapsible part of the airway is composed of numerous muscles and soft tissue in order to perform functional tasks such as speech, swallowing of food/liquids, and the passage of air for breathing, but lacks rigid or bony support (Malhotra and White, 2002; Patil et al., 2007b; Eckert and Malhotra, 2008). During sleep, all muscles tend to relax, including the UA muscles. In healthy subjects, muscle tone is high enough to prevent the UA from collapse during sleep (Sands et al., 2014). In patients with the diagnosis of obstructive sleep apnea (OSA), the UA muscles are not capable to maintain UA opening due to a combination of anatomical and non-anatomical reasons, resulting in recurrent episodes of complete or partial collapse. This manifests as a reduction (hypopnea) or complete cessation (apnea) of airflow lasting at least 10 s. The apnea/hypopnea index (AHI) is a measure of the severity of OSA and is calculated as the number of apneas and hypopneas per hour of sleep. The diagnosis of sleep apnea is confirmed if the AHI is ≥ 5 events per hour of sleep. Based on the AHI, the following levels of severity are defined: mild (5 ≤ AHI < 15/h), moderate (15 ≤ AHI < 30/h), and severe (AHI ≥ 30/h) sleep apnea (American Academy of Sleep Medicine Task Force, 1999). The prevalence of OSA is increasing, and it is estimated that 14% of men and 5% of women suffer from the disease (Peppard et al., 2013).

Undiagnosed and/or untreated OSA leads to daytime sleepiness, motor vehicle accidents, and diminished quality of life (Young et al., 2002; Dempsey et al., 2010). Furthermore, OSA is associated with an increased risk for arterial hypertension, stroke, and cardiovascular comorbidity and mortality (Young et al., 2002; Marin et al., 2005; Dempsey et al., 2010).

In order to reduce these comorbidities, a successful treatment is important (Marin et al., 2012; Potts et al., 2013). The choice of treatment for OSA is dependent on the severity of the disease, UA anatomy and the preference of the patients (Epstein et al., 2009). Continuous positive airway pressure (CPAP) is the gold standard treatment for patients with moderate to severe OSA (Sullivan et al., 1981; Young et al., 2002). However, suboptimal and occasionally poor adherence and acceptance is a limitation of CPAP therapy and therefore, can lead to less potential benefits of the therapy (Rotenberg et al., 2016). In these patients, alternative treatment options need to be considered. Oral appliance (OA) therapy is a non-invasive treatment option in patients with mild OSA or in patients with moderate to severe OSA who do not tolerate, do not accept or refuse, or do not comply with CPAP therapy (Sutherland et al., 2014). In addition, a variety of surgical methods can alternatively be used to treat OSA. Surgical therapy includes bypass procedures (tracheostomy), nasal reconstruction and UA surgery techniques that modify the soft tissue surrounding the oropharynx or hypopharynx either by tissue reduction or stabilization and advancement (Beyers et al., 2016; Doghramji and Boon, 2016; Vanderveken et al., 2016). Some examples of such UA surgery techniques are uvulopalatopharynogplasty, palatal implants, tongue advancement (e.g., hyoid suspension) or reduction (e.g., glossectomy), and maxillomandibular advancement surgery (Epstein et al., 2009; Beyers et al., 2016). The acceptance of these techniques is limited by the side effects and the lack of resilient data on effectiveness (Caples et al., 2010).

**Figure 1:**
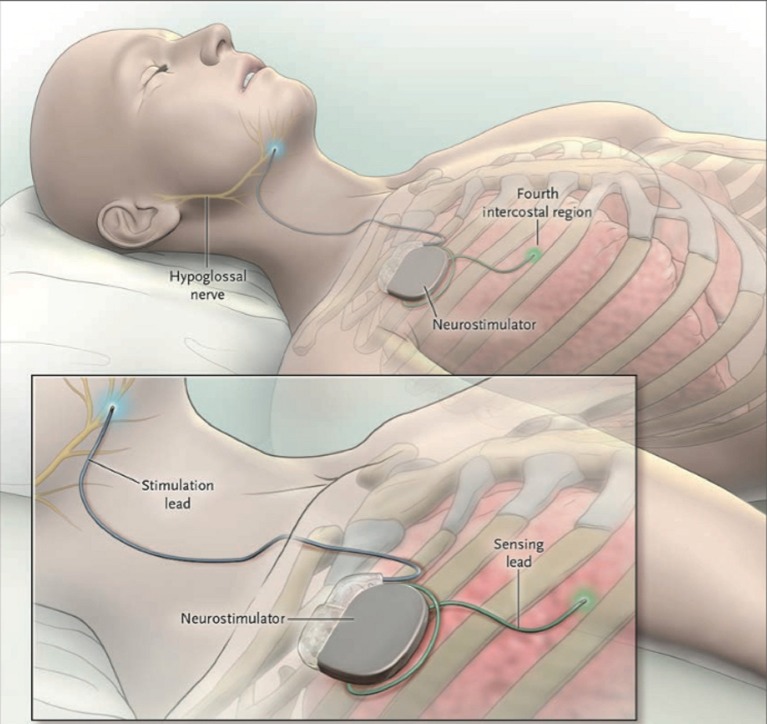
The Inspire II Upper Airway Stimulation (UAS) device. Three different parts are implanted in order to give electrical stimulation pulses to the hypoglossal nerve. The sensing lead detects in- and expiration of the patient during sleep. After conversion of the respiratory signal by the neurostimulator, stimulating pulses are delivered to the hypoglossal nerve through the stimulation lead. This means that a unilateral respiration-synchronized stimulation of the hypoglossal nerve generates a protrusion of the tongue. From Strollo et al. (2014), Copyright © Massachusetts Medical Society. Reprinted with permission.

Electrical neurostimulation therapy of the hypoglossal nerve (n. XII) is an emerging treatment option with clinical results published on three different systems, each offering a different type of working mechanism: the Hypoglossal Nerve Stimulation (HGNS) system (Apnex Medical, Inc., St. Paul, Minnesota) (Eastwood et al., 2011), the Aura6000 Targeted Hypoglossal Neurostimulation (THN) system (ImThera Medical, Inc., San Diego, California) (Friedman et al., 2016), and the Inspire II Upper Airway Stimulation (UAS) device (Inspire Medical Systems, Inc., Maple Grove, Minnesota) (Vanderveken et al., 2016). This paper will further focus on the Inspire UAS system, shown in Figure 1, being the only system having Food and Drug Administration (FDA) approval at this stage.

UAS therapy addresses the reduced activity of the UA dilator muscles during sleep without altering its anatomy. It has the ability to provide a multilevel effect on the UA collapsibility with one procedure (Safiruddin et al., 2015). During this treatment, a protrusion of the tongue is generated by a unilateral respiration-synchronized stimulation of the n. XII. The potential of hypoglossal nerve stimulation using the Inspire I stimulating system was first proven by Schwartz et al. in a clinical trial with eight patients (Schwartz et al., 2001). In the following years, adjustments and improvements to the system were made. In addition, patient selection became very important to predict treatment outcome. The effectiveness and adherence of the current UAS device are well-documented (Van de Heyning et al., 2012; Strollo et al., 2014; Heiser et al., 2017). However, selecting the most suitable patients for UAS therapy and setting up the right therapeutic tongue stimulation are complex processes, making it challenging for implementation in a clinical pathway. Clinical pathways deal with a high volume of complex care processes in a structured way and aim to improve the quality of healthcare (Pearson et al., 1995; Deneckere et al., 2011, 2012; Vanhaecht et al., 2012).

In 2014, the first results of the Stimulation Therapy for Apnea Reduction (STAR) trial, a prospective multi-center trial, were published using the Inspire II UAS device (Strollo et al., 2014). The results of this study were promising. Serious adverse events were uncommon; side effects did not bother the patients or were resolved after habituation of the therapy. In addition, a randomized withdrawal study was conducted in the responders of this study, showing continuous stimulation is needed to maintain adequate response. Therapy withdrawal led to an increased OSA severity, comparable to pretreatment values (Strollo et al., 2014; Woodson et al., 2014). The promising results of the STAR trial led to US FDA approval for the Inspire II UAS device in April 2014. Recently, the 4-year follow-up data of the STAR trial results were published, showing stable effects on primary and secondary outcome parameters up to 4 years after implantation of the UAS neurostimulator (Gillespie et al., 2017).

The aim of this paper is to describe the mechanism of action of UAS in function of OSA pathophysiology, the current surgical technique, and, to introduce and share a clinical pathway for UAS therapy for OSA patients.

## Materials and methods

### Patient selection

The diagnostic workup and patient selection for UAS therapy starts with a medical out-patient clinic visit, involving medical history taking and assessment of anthropometric parameters. The main anthropometric parameter of importance is obesity: severe obesity increases the risk for OSA (Mortimore et al., 1998; White, 2005; Kirkness et al., 2008). More specifically for UAS candidates, the body mass index (BMI) should be below 32 kg/m^2^ in order to be eligible for UAS therapy (Van de Heyning et al., 2012). Other clinical exclusion criteria include neuromuscular disorders, like n. XII palsy, severe cardiopulmonary disorders, active psychiatric disease, and comorbid non-respiratory sleep disorders, as all these disorders may bias treatment mechanisms or sleep assessment (Ong et al., 2016).

Polysomnography (PSG) is a second step in the patient selection procedure for UAS. The AHI, a marker of OSA severity, is used as criterion for in- or exclusion: AHI is recommended to be between 15 and 65/h (Strollo et al., 2014; Ong et al., 2016; Vanderveken et al., 2016). Other sleep parameters include the percentage of central apneas, which is preferred to be below 25%. Central sleep apnea is, in general, caused by the combination of an unstable ventilatory control system and a high hypercapnic responsiveness (White, 2005; Verbraecken and De Backer, 2009). Both these factors are non-anatomical factors, which might possibly not be directly altered by UAS.

A third and final step in the diagnostic workup and patient selection procedure is to investigate the UA collapsibility. In our hospital, this is done by performing a so-called drug-induced sedation endoscopy (DISE). During DISE, a nasendoscope is inserted via the nose into the UA (Rojewski et al., 1984; Vanderveken et al., 2013; Vroegop et al., 2014; Viana et al., 2015; Ong et al., 2016). Sedatives, usually midazolam and/or propofol, are used to mimic natural sleep in patients (Hohenhorst et al., 2012; Vanderveken et al., 2013). Each collapse is scored based on the site of collapse (palate, oropharynx, tongue base, hypopharynx, or epiglottis), degree of upper airway collapse (partial or complete) and the pattern of collapse (anteroposterior, laterolateral, or concentric). In a majority of patients, the UA collapse is not limited to one region, and a multilevel collapse is present (Vroegop et al., 2014). The severity of UA collapsibility can also be estimated by determining the pharyngeal critical closing pressure (Pcrit). The active Pcrit is measured by slowly lowering the nasal pressure to different subtherapeutic pressure levels using CPAP and subsequently, the nasal pressure is plotted against the associated peak flow (Gold and Schwartz, 1996; Boudewyns et al., 2000; Azarbarzin et al., 2017). On the other hand, the passive Pcrit is measured by abruptly lowering the pressure and measuring the flow before activation of the muscles (Patil et al., 2007a).

Some collapse patterns or anatomical observations are considered as contraindications for UAS. A first contraindication is the presence of large tonsils. These large tonsils usually result in a laterolateral collapse of the pharynx. By moving the tongue forward with UAS, it is unlikely that this laterolateral collapse will be resolved. However, these large tonsils can be removed in advance via tonsillectomy. Another contraindication for UAS that can be observed during DISE is the presence of a complete concentric collapse at the level of the palate (Vanderveken et al., 2013; Vroegop et al., 2014; Ong et al., 2016). These patient selection criteria were developed based on early feasibility studies (Van de Heyning et al., 2012) and validated in later larger trials (Strollo et al., 2014).

### The UAS system

**Figure 2:**
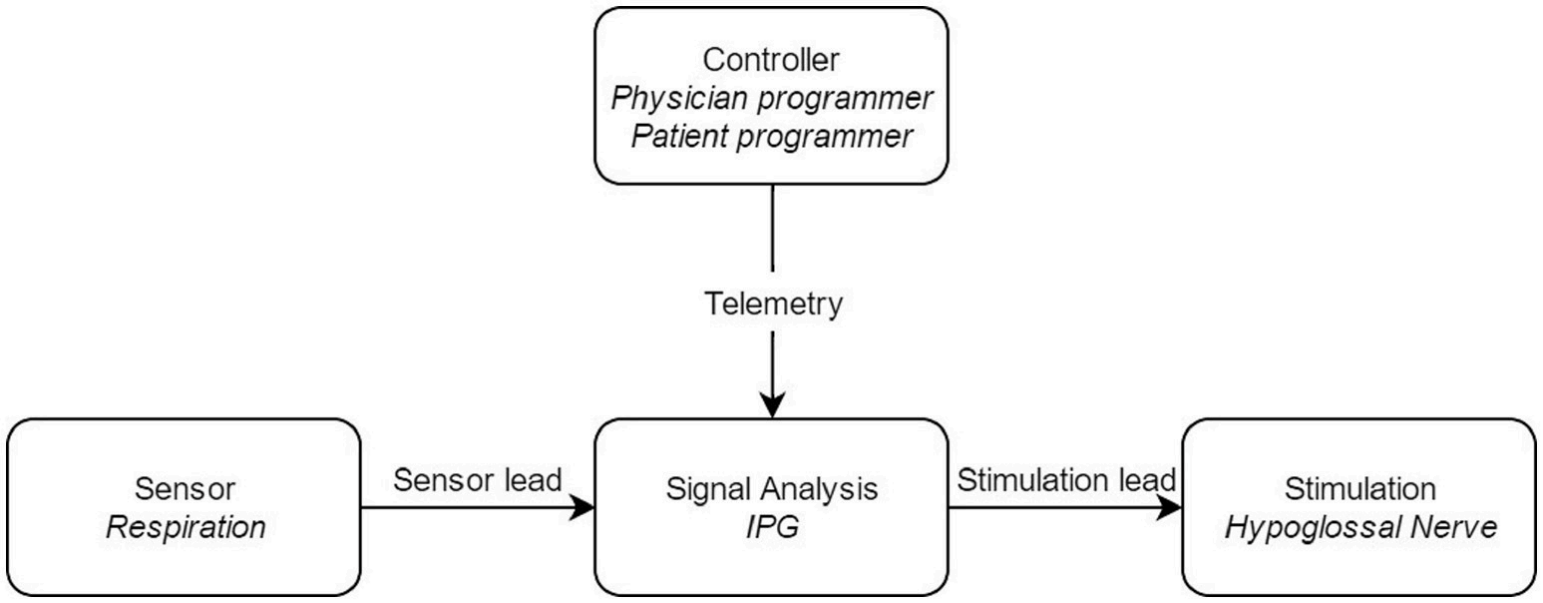
Block diagram of the upper airway stimulation, with the different components of the system. IPG: Implantable Pulse Generator.

**Figure 3:**
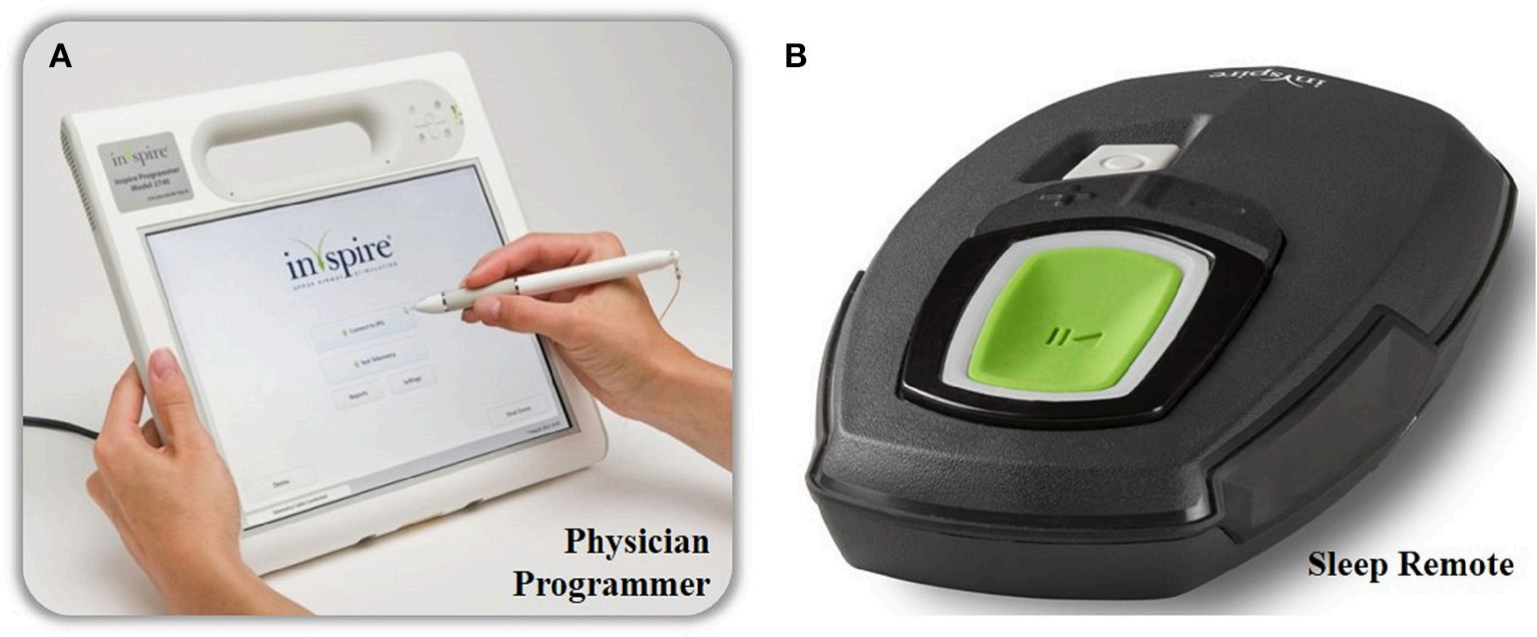
The external components of the Inspire II UAS device. The physician's programmer is shown on the left **(A)** and is used during the implant surgery, activation and in-hospital titration night. With the physician's programmer, more advanced adjustments are made. The patient's programmer is shown on the right **(B)** and is used by the patient to turn on and off therapy and for small adaptions in stimulation amplitude.

The UAS device consists of five distinct parts, three implantable and two external components, interconnected via either leads or telemetry (see Figure 2). The components that need to be implanted during the surgical procedure are the implantable pulse generator (IPG), the stimulation lead and the sensing lead (see Figure 1). Both external UAS components, the patient's programmer and the physician's programmer, should be regarded as controllers (see Figures 2, 3).

The sensing lead with the accompanying sensor is used to “sense” the breathing pattern and synchronize the n. XII stimulation with breathing (Strollo et al., 2014). One side of the sensor consists of a piezoelectric crystal mounted inside a sealed housing. The sensor is placed between the fifth and sixth rib in a tunnel of ~6 cm in length between the external and internal intercostal muscles (Heiser et al., 2016b). As only one side of the sensor is made from piezoelectric material, the sensor has to be implanted with the piezoelectric material facing the pleura (Strollo et al., 2014; Heiser et al., 2016b). To avoid movement and revolving of the sensor, the sensing lead is slightly flattened. Flatter surfaces will increase friction due to the larger surface area, resulting in a fixed sensor (Christopherson et al., 2011). In addition, by coating the lead tip with a thinned, medically approved adhesive, the sensor is fixed more firmly. Another advantage of this coating is a reduction in sensor lead friction, reducing possible tissue damage (Christopherson et al., 2011). The actual detection of the inspiration and expiration pattern is done by the pressure sensor. During inspiration, the pressure becomes negative. On the other hand, pressure will be positive during expiration. By using the piezoelectric crystal, the pressure differences are converted into electric current, which acts as the sensing signal.

The sensing signal is transferred to the IPG, in which it is processed by filtering operations. Especially cardiac artifacts should be filtered out (Doyle et al., 2003; Christopherson et al., 2011). The IPG has a double function. It contains the battery and couples the sensed respiration to the stimulation (Van de Heyning et al., 2012). Inside the IPG, the sensed breathing signal is bandpass filtered, amplified, normalized, and its amplitude sampled (Doyle et al., 2003). Each amplitude sample is compared to programmed thresholds for inspiration onset and offset. If the sample amplitude exceeds the onset threshold, stimulation is started. Once the offset threshold is detected, stimulation is turned off. As respiration is highly stable during sleep, a refraction period can be preset after stimulation offset detection. The refraction period will avoid overstimulation, and will reduce the effect of movement artifacts (Doyle et al., 2003).

**Figure 4:**
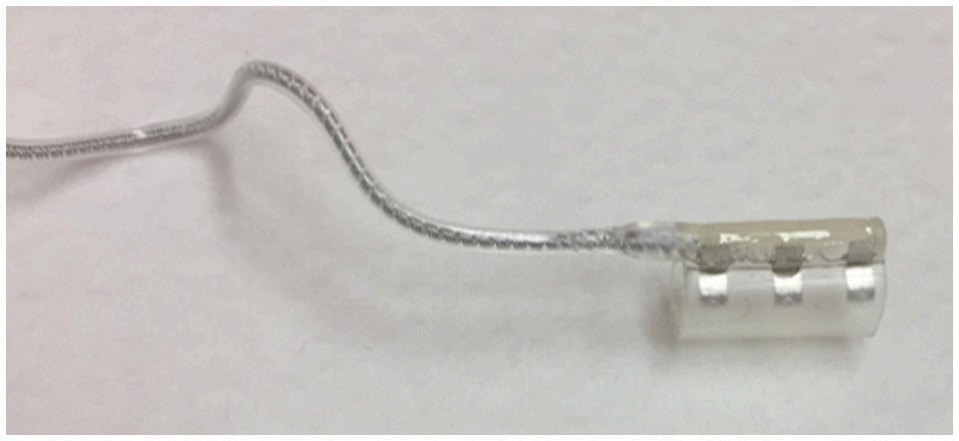
The stimulation cuff in which three stimulation electrodes are embedded. The stimulation cuff is placed around the protruding branches of the hypoglossal nerve.

The stimulation lead connects the IPG to the cuff electrode, which is placed around the n. XII. The electrical stimulation excites the n. XII. Depolarization is achieved via an ion flow through the neuronal cell membrane, which in turn will generate an action potential. (Tsui, 2008). This depolarization will in turn excite the genioglossus muscle, resulting in tongue protrusion. The stimulation is triggered via three stimulation electrodes, embedded inside a stimulation cuff (see Figure 4). To only include protruding branches of the nerve, avoiding stimulation of the retractor muscles, the m. hyoglossus and the m. styloglossus, only the medial site of the nervus hypoglossus is included in the stimulation cuff. Selective nerve monitoring can be used for identification and differentiation of the branches causing protrusion and retraction of the tongue. Therefore, the precise inclusion and exclusion of nerve branches during intraoperative placement of the stimulation cuff is facilitated (Heiser et al., 2016a). If possible, also the C1 nerve is included inside the cuff as this nerve supplies the geniohyoid muscle, the second protruding muscle (Heiser et al., 2016a). Stimulation of both nerves will cause tongue protrusion, opening the UA (Van de Heyning et al., 2012; Heiser et al., 2016a,b).

By using a programming unit consisting of a physician programmer and a patient programmer (see Figure 3), the IPG can be titrated and the stimulation parameters can be optimized via short-range radiofrequency telemetry (Maurer et al., 2012). The patient programmer is used by the patient to switch the stimulation on and off and to make small adjustments in stimulation amplitude, within personal preselected limits (Maurer et al., 2012).

On the other hand, the physician programmer is a tablet that is connected to the IPG telemetry unit via Bluetooth and is used for a non-invasive read-out and to optimize stimulation and sensing parameters (Maurer et al., 2012).

### Implantation of the UAS system—the surgical procedure

The implantation of the UAS system is performed under general anesthesia via nasal intubation. The operative technique and different steps in the procedure are well-described by Heiser et al. (2016b). In short, the procedure starts with positioning of the patient: the neck of the patient is extended and turned to the left. Towel rolls or positioning pillows are used for a good placement of the patient. A nerve integrity monitoring system (NIM 3.0, Medtronic Xomed, Jacksonville, FL) is used during the implantation procedure to decide which branches can be included in the stimulation cuff electrode and which branches need to be excluded (Heiser et al., 2016a). One electrode of the monitoring system is placed in the right anterior floor of the tongue, directed in a vertical direction just posterior to the mandible. This first electrode has the purpose to monitor the genioglossus muscle for the inclusion of branches in the cuff. For the exclusion of branches, a second electrode to monitor the m. styloglossus and m. hyoglossus is placed along the ventrolateral aspect of the right tongue in a posterior direction, just underneath the mucosa. During the implantation of the device, the tongue and tongue movement can be visualized by covering the mouth with a transparent coating.

Once the patient is carefully positioned, the real implantation procedure can start. Three incisions are made during the procedure.

The first incision, that is made in the right submandibular and submental neck region, is used for the placement of the stimulation lead and measures about 3–5 cm. This incision starts ~1 cm to the right of the midline and goes to the anterior edge of the submandibular gland. The incision is situated about one finger breadth below the mandible. Once the n. XII main trunk is identified, the retraction branches can be separated from the protrusion branches. The latter will be included in the cuff electrode. The nerve integrity monitoring system is used to verify the separation of the nerve fibers. The cuff electrode is placed around the protrusion branches and the lead is then anchored on the lateral aspect of the digastric tendon.

A second incision is made at the right anterior chest wall, about 3–4 cm inferior of the midway along the clavicle and is about 5 cm long. This incision is used to make a pocket, in which the IPG is positioned. The pocket should have a dimension of ~5 by 6 cm, in order to make sure the IPG will fit in the pocket.

A third and last incision is made horizontally along the lateral chest, in the fifth or sixth intercostal space, and is used for the placement of the sensor lead. It is 5 cm in length with the lateral extent being the middle of the axilla and the medial extent the inferolateral border of the m. pectoralis major. A tunnel of ~6 cm is made between the external and internal intercostal muscles. Next, the sensor and stimulation leads are tunneled to the pocket of the IPG and the three components and the leads are interconnected.

**Figure 5:**
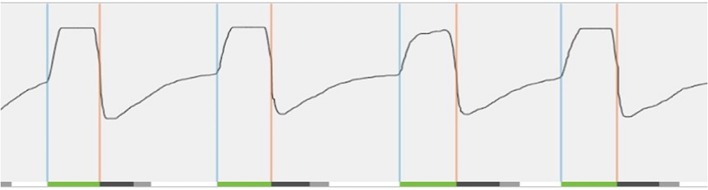
Real-time sensor waveform test during implantation. The respiration and stimulation are shown in the graph. During breathing, a change in pressure is detected by the sensor. This information is sent to the implantable pulse generator. Stimulation (green line at bottom) is triggered (blue vertical line) and should last until expiration is detected (orange vertical line). In order to prevent stimulation immediately after exhalation, the off period begins (dark gray line at bottom). After the off period, a new stimulation can be triggered. When this does not occur immediately, there is a period without stimulation (light gray line at bottom).

If all components are implanted and connected, the device will be validated by testing the sensor waveform (see Figure 5) and by running stimulation, in order to visualize tongue movement.

### Mechanism of action: engineering aspect

After the implantation of the UAS device, several parameters can be individually optimized and titrated. The parameters that can be optimized include the electrode configuration, the stimulation amplitude, and the stimulation pulse width and rate.

**Figure 6:**
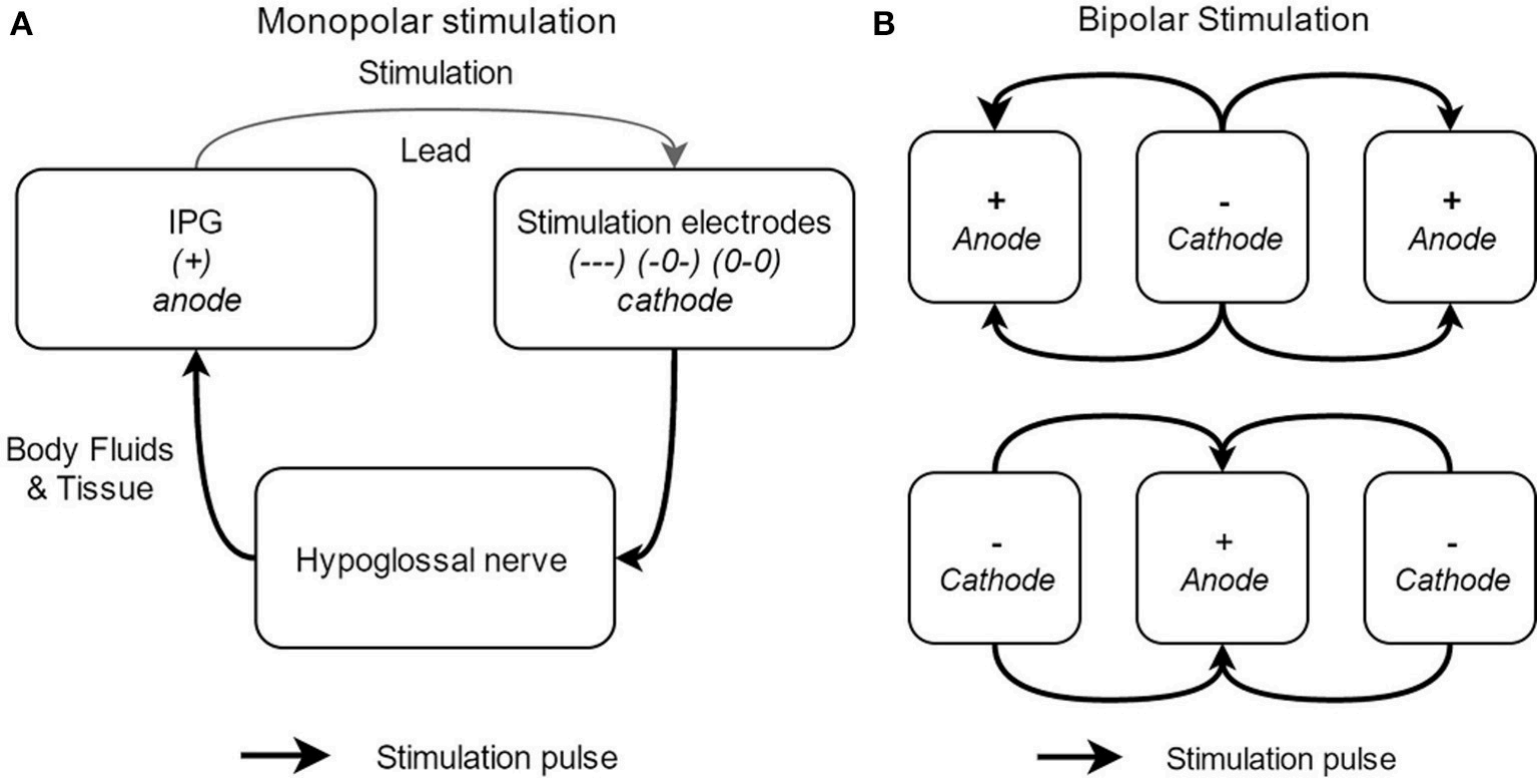
Schematical overview of the different electrode stimulation configurations, with the monopolar stimulation **(A)** and the bipolar stimulation **(B)**.

In general, two different electrode configurations are reported: monopolar (Figure 6A) and bipolar (Figure 6B) configurations. The so-called “standard” configuration is the bipolar (+-+) configuration (see upper part of Figure 6B). In this electrode configuration, the stimulation current loop is made inside the cuff and the IPG is inactive (OFF). This leads to a highly local current loop, avoiding stimulation of adjacent nerves or nerve branches. Four alternative electrode configurations are provided by the Inspire UAS device: one other bipolar configuration (-+-) and three monopolar configurations (—), (0-0), and (-0-). In the latter, monopolar, case, the IPG functions as the anode and the cuff electrodes as the cathode. In this configuration, the stimulation impulse flows through the cathode, stimulates the n. XII, and then returns via body fluid and tissue to the anode, creating a closed current loop (see Figure 6). The monopolar configuration causes a deeper current penetration and wider electric field (between the cuff and the IPG) without a higher stimulation amplitude (Heiser, 2016). The three monopolar configurations differ in the applied current intensity and current density. More (–) activated electrodes will result in a larger possible current intensity and a higher current density (Tsui, 2008). In other words, the pattern of n. XII capture can be affected by alternative electrode configurations. This means that the depth of the stimulation will be adapted as well as the vessels of the nerve that are stimulated by the cuff electrodes.

A second parameter that can be titrated and optimized is the stimulation amplitude. The required stimulation amplitude is highly distance dependent (Tsui, 2008). As n. XII stimulation uses a cuff electrode placed directly around the n. XII, the current amplitudes required are rather low. However, if it was impossible to include the C1 nerve in the cuff electrode during the surgical implantation procedure, higher stimulation amplitudes might increase stimulation efficacy by stimulation of this adjacent C1 nerve (Heiser, 2016). Despite this possible rise in efficacy, the stimulation amplitude should be kept as low as possible to avoid sleep arousal or even nerve or tissue damage.

Finally, in an advanced titration process, the pulse width and rate can be adapted. Stimulation consists of a burst of pulses and by changing the duration of the pulses (μs), the gross strength can be adjusted. Together with changing the pulse width, the electrical field that is generated will change. In order to maintain the same electric field, the rate (pulses per second [Hz]) can be increased or decreased. When the tongue is protruding too fast or too slow, it is recommended to adapt the pulse width and the rate in order to control the smoothness of the stimulation sensation. In general, the difference on stimulation due to its pulse width is distance-dependent. The further away from the nerve, the greater the pulse width effect. Smaller pulse widths require higher current amplitudes (Tsui, 2008). The pulse width should always be large enough to reach chronaxie, the minimum stimulation time required to induce stimulation at two times rheobase amplitude, or the minimum intensity needed for stimulation.

### UAS clinical pathway

**Figure 7:**
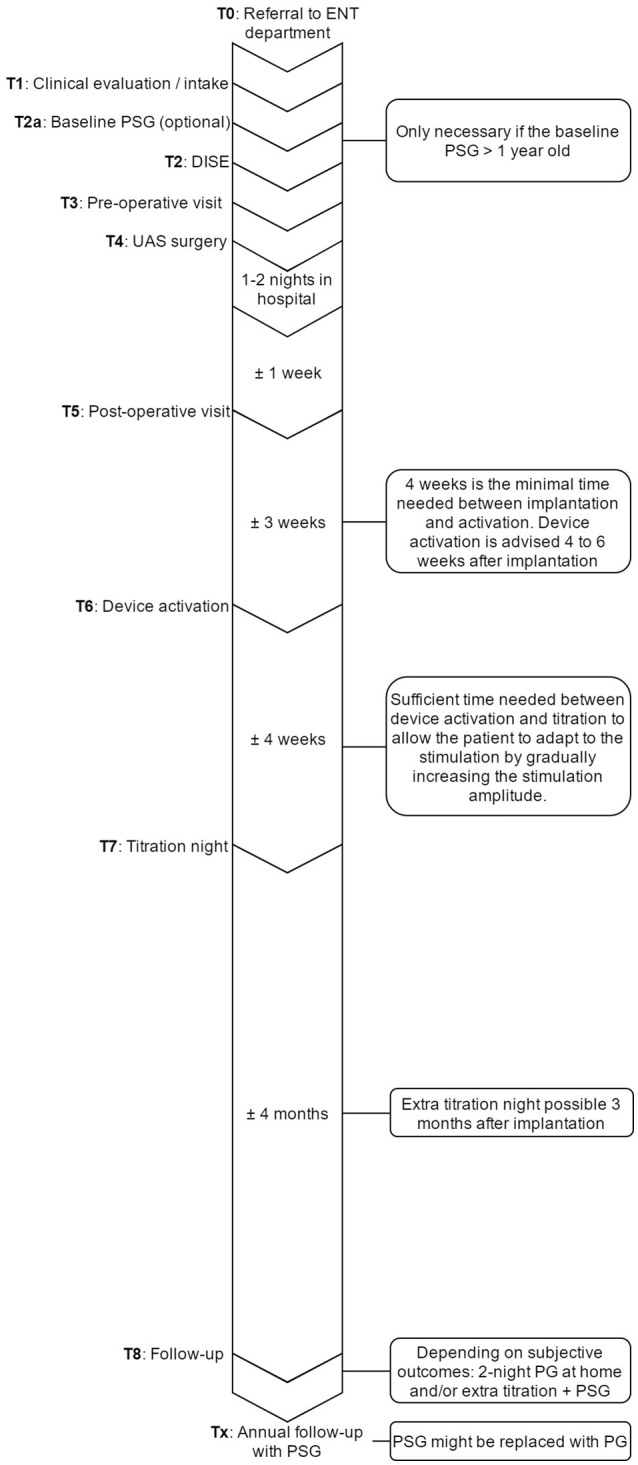
Overview of the UAS pathway that provides an outline of the steps that are undertaken in the care process and their sequence in time. ENT, ear, nose, and throat department; PSG, polysomnography; UAS, upper airway stimulation; DISE, drug induced sedation endoscopy; PG, polygraphy.

Clinical pathways are used to organize complex care processes by providing structure and standardization. The multidisciplinary approach of UAS therapy and the different investigations needed in the diagnostic workup and patient selection, together with the need for post-operative titration and strict clinical follow-up make it suitable for such a structured pathway approach. Based on scientific evidence as well as personal experience, the clinical pathway was developed. The individual steps and their sequence are shown in Figure 7. The most critical steps within the UAS clinical pathway are described in detail.

T0 Inclusion in the UAS clinical pathway starts at the referral to the Ear, Nose, Throat (ENT), head, and neck surgery department from various physicians within the multidisciplinary sleep medicine team at the Antwerp University Hospital or from external referral.

T1 Before inclusion, if there is no contraindication from a medical point of view for UAS therapy, patients need to undergo a DISE. Furthermore, it is determined whether or not the PSG needs to be repeated. The PSG can be maximum 1 year old and has to be a baseline measurement, meaning that patients must discontinue any other treatment, like CPAP or OA. Treatment with CPAP must be suspended for at least 10 days in advance (Vroegop et al., 2015). If the PSG needs to be repeated, it will preferably be scheduled the night before the DISE.

T2 For the DISE, patients will be hospitalized at the one day clinic. The DISE is used to clinically and dynamically investigate the UA during artificial sleep and is a promising technique for the selection and prediction of treatment success for non-CPAP treatment options (Eichler et al., 2013; Vanderveken, 2013; Vanderveken et al., 2013). Midazolam with a bolus injection of 1.5 mg and/or propofol, using a target-controlled infusion system at a target of 2.0 to 3.0 μg/mL, is used to induce sedation. The level, the direction and the degree of UA collapse can be assessed, and a prediction about the effectiveness of UAS can be made based on the behavior of the UA (Hewitt et al., 2009; Hamans et al., 2010; Kezirian et al., 2011; Vanderveken et al., 2011, 2013; Vroegop et al., 2013).

Based on previous research and success rates in the past (Van de Heyning et al., 2012; Strollo et al., 2014), the inclusion criteria for UAS were set. Patients with a history of moderate to severe OSA who are non-compliant to CPAP treatment are eligible for screening. Patients with a BMI < 32 kg/m^2^, an AHI between 15 and 65 events/h, no central sleep apnea (<25% of total AHI) and without a complete concentric collapse at the palate during DISE are qualified for UAS-therapy (Vanderveken et al., 2013).

T3 Before the day of the implantation, patients have to come to the hospital for an out-patient visit with the anesthesiologist and the surgeon in order to make sure the whole procedure is clear and no more questions remain. This visit with the anesthesiologist and the ENT, Head, and Neck surgeon also includes the finalization of the formal anesthesia and surgical informed consent, respectively.

T4 The surgery is performed under general anesthesia via nasal intubation, as described above. During surgery, the UAS device is implanted and a device check to test the different individual components is performed. A chest X-ray is obtained after surgery to rule out an ipsilateral pneumothorax. After a stay of 1–2 nights in the hospital, patients are discharged. Wound care instructions are provided.

T5 ~1 week after the implantation, the post-operative visit is scheduled for history taking and clinical examination performed by the ENT, Head, and Neck surgeon. During this visit, sutures are removed, but the device is not yet activated.

T6 The activation of the device will take place during an out-patient visit, ~1 month after surgery. For the activation, the standard settings (electrode configuration (+–+), pulse width 90 μs, frequency 33 Hz, max stimulation time 4 s, exhalation −4/−1, inhalation 0/+1, off period 38/13) are used, while different tests are performed to assess the functioning and to program the patient-specific parameters. A first test evaluates the stimulation thresholds, which are the sensation threshold (lowest amplitude the patient can feel the stimulation), functional threshold (lowest amplitude the tongue just passes beyond the lower incisors), and sub-discomfort threshold (highest amplitude the patient feels comfortable). Furthermore, impedances are measured and running a waveform tests the sensory lead. During the waveform run, a real-time sensor waveform can be seen (see Figure 5). The respiration and the stimulation are shown in a graph. A change in pressure occurs during breathing, which is detected by the sensor, and this information is sent to the IPG. When inspiration starts, stimulation is triggered and should last until expiration is detected. When the stimulation stops, the off period begins in order to prevent stimulation immediately after exhalation. When inspiration is detected again, a new stimulation will be triggered. The patient receives a remote control device with instructions how to use it at home. Stimulation starts at functional threshold to minimize the effect of the device on sleep quality. Usually, 11 stimulation amplitude steps (functional threshold + 1.0 volts) are given to the patient. In this way, he has the ability to gradually increase the strength of the stimulation at home after a range of 3 days, awaiting the in-hospital titration night (T7), and to adapt to the stimulation and the use of the device. The “all night, every night” usage is encouraged, since it is critical for acclimation and long-term adaption of the therapy.

T7 An in hospital titration night is carried out during a full-night attended PSG about 1 month after the activation and thus 2 months after surgery. During this titration night, a PSG is conducted while the device is adjusted to the patient until respiratory events are eliminated. A programming device modifies the settings of the implant. Before the start of the sleep study, the same tests are performed as during the activation (T6), but this time, different electrode configurations are considered to optimize the control of OSA and patient comfort. Furthermore, the tongue movement is evaluated. During the titration night, amplitude is the primary stimulation strength parameter that is adjusted. Increasing the amplitude is how the therapy is titrated to minimize the occurrence of obstructive events. The titration needs to be slow in order to give the airway and the entire respiration system time to stabilize following obstructive events. After the parameters are set, the patients can utilize their device using these settings within a narrow range (5 steps: therapeutic amplitude ± 0.2 volts). The final patient's amplitude should minimize the occurrence of obstructive events, the lower limit of the control range should maintain the therapeutic effect and the upper limit should not exceed arousal threshold. Patients can increase or decrease the voltage within this range to maximize the positive effect on sleep and snoring and to mitigate discomfort.

T8 6 months after implantation, the patients receive a phone call to check their status. If the patient indicates he does not feel better, a second or advanced titration night is scheduled. If the patient is subjectively feeling better, a validated two-night portable sleep monitor measurement is performed at home in order to objectify the treatment progression. Based on these results, an advanced titration or other further action, such as combination therapy with OA or sleep position trainers, will be taken if necessary (Lee et al., 2015). Once treatment is successful, patients will be followed-up on annual basis for a PSG and a device check. During the latter, the battery status, usage and current settings are evaluated and the stimulation thresholds are tested.

## Discussion and conclusions

OSA is a common disorder with different treatment options going from non-surgical approaches to a wide variety of surgical procedures (Kezirian and Goldberg, 2006; Randerath et al., 2011). For treatment of OSA without the use of CPAP, UAS can be considered as a good alternative (Kezirian et al., 2010; Van de Heyning et al., 2012; Woodson et al., 2016; Heiser et al., 2017).

Clinical evidence for UAS treatment was shown first in the multi-center STAR trial including 126 patients (Strollo et al., 2014). Over a 12 month follow-up period, the AHI and oxygen desaturation index (ODI) both decreased significantly (*p* < 0.0001) with a reduction from 29.3/h to 9.0 events/h and a reduction from 25.4 to 7.4/h respectively. Also secondary outcomes, measured by the Epworth Sleepiness Scale (ESS) and Functional Outcomes of Sleep Questionnaire (FOSQ), showed improvements in quality-of-life-measures. These results were confirmed in the 18, 24, 36, and 48 months follow-up (Strollo et al., 2015; Soose et al., 2016; Woodson et al., 2016; Gillespie et al., 2017). After therapy withdrawal, AHI and ODI measures returned to pre-treatment values.

Clinical evidence established in the STAR trial was confirmed in several single- and multicenter studies. Two single-center studies have shown significant AHI reductions and significant improvements on ESS without reporting any serious adverse events (Kent et al., 2016; Heiser et al., 2017). Adherence in both studies was high (7.0 ± 2.2 h per night and 6.6 ± 2.7 h per night respectively). Thaler et al. reported surgical cure (AHI post-implant < 10/h) in all eight subjects included in this case series (Thaler and Schwab, 2016). Also in this study, no serious adverse events were reported. Recently, Heiser et al. published the results of a multi-center German post-market study (Heiser et al., 2017). Significant improvements were found in AHI, ODI, minimal oxygen saturation (SpO_2_), ESS and FOSQ outcomes 6 months post-implantation in 60 patients. These results were confirmed after 12 months (Steffen et al., 2017). The THN system was studied by Mwenge et al. in which participants showed clinically relevant improvements in objective and subjective measurements (Mwenge et al., 2013). More recently, these results were confirmed in the study of Friedman et al. (2016). In this study, it was suggested that THN therapy in selected patients is likely to be safe and effective. It can thus be concluded that electrical stimulation of the n.XII is a promising treatment option in patients with CPAP-failure.

One of the most important factors in order to achieve treatment success in UAS is patient selection, as described by Van de Heyning et al. (2012). The study of Van de Heyning et al. was divided in two different parts, where patient selection of the second part of the study was based on the positive predicting factors emerging from the first study. In the second part of the study, a significant improvement in treatment outcome was achieved. In another study of Vanderveken et al. the importance of DISE as a selection tool for UAS was confirmed (Vanderveken et al., 2013). The results indicated that the absence of a complete concentric collapse at the level of the palate could be predictive for treatment success for OSA patients with implanted UAS therapy. Patients must fulfill the criteria listed in Table 1 in order to be eligible for UAS implantation. Pcrit measurement is another method to estimate the UA collapsibility. In a recent study of Azarbarzin et al. it was demonstrated that it is feasible to estimate the UA collapsibility by using peak flow in OSA patients taking into account that the peak flow at zero nasal pressure captures the essence of the collapsibility (Azarbarzin et al., 2017). Therefore, Pcrit might be used to select patients by calculating the UA collapsitility in an automatic, minimally invasive manner.

**Table 1:** Most important inclusion and exclusion criteria for UAS implantation.

**Inclusion**	**Exclusion**
CPAP noncompliant and/or intolerant	ccc at soft palate
15/h < AHI < 65/h	central sleep apnea > 25%
BMI < 32 kg/m^2^	severe comorbidities

*CPAP, continuous positive airway pressure; AHI, apnea/hypopnea index; BMI, body mass index; ccc, complete concentric collapse*.

The Inspire system consists of different parts, which may or may not be implanted (Figures 1–3). All parts are connected in order to have an optimally working system (Figure 2). Each part has its own function. A thorough surgical procedure is of utmost importance to ensure the correct placement of the different parts of the device. The placement of the cuff around the protruding branches of the n. XII can differ in patients, since every patient is unique. An update of this procedure is well-described by Heiser et al. and in our hospital, surgery is based on this step-by-step implant procedure (Heiser et al., 2016b). A standardized operative technique is helpful in achieving consistent therapy outcomes.

Besides the surgical procedure, treatment outcome is dependent on the different parameters of the device. Consequently, the parameters of the device are patient specific and need to be optimized for each patient individually. By changing electrode configuration, stimulation amplitude, and stimulation pulse width and rate, improvement in treatment outcome can be obtained for each patient. The optimization needs to be done at night, while conducting a PSG, a so-called UAS titration night. During the PSG, the lab technician changes the parameters until the best patient specific settings are reached. Due to the variation in parameters, the device is adaptable. Therefore, better treatment outcomes can be obtained and patient specific needs can be fulfilled. The fact that the parameters can be changed for each patient individually is thus a major advantage of the device.

The multidisciplinary approach of UAS treatment and the different steps needed in the treatment plan to optimize the therapy, makes it suitable for implementation in a structured pathway approach. This clinical pathway includes a standardized (diagnosis) and patient specific (therapy and follow-up) part. Even though the actual procedures in the care process did not change, the delivery of patient care became more efficient in terms of time by optimizing the triage and sequence of the investigations, and with the members of each team focusing on their particular part of the clinical pathway. By taking the vision of each individual team member into account, a better strategy to reorganize the care process toward excellence can be achieved. The positive effect on the delivery of patient care is mainly due to the fact that all screening investigations take place on the same day (T2). Furthermore, from the moment the patient is eligible according to inclusion criteria and has agreed to undergo surgery, all time slots are pre-assigned. These schedules may vary, since they are based on the patient's availability.

Pathways can be used for complex interventions and should not be defined as documents or tools. They work best when tailored to local rather than generic contexts (Craig et al., 2008; Lodewijckx et al., 2012; Vanhaecht et al., 2012). For UAS therapy, the diagnostic work-up, patient selection and activation of the therapy are processes that are strongly contoured care blocks. However, the therapeutic follow-up is more variable. For example, if we compare the clinical pathway that is used in our center with that used in the center of Heiser et al., the diagnostic work-up, activation, and initial titration night of the UAS device is very comparable. However, in the center of Heiser et al. (2017), a second titration night is performed in every patient during a second PSG 3 months after the implantation. Furthermore, in that center, a home sleep polygraphy is performed at 6 and 12 months follow-up. Whereas, in our center, after the initial titration, the patient is called 4 months later, to check their status. A second titration night is only performed when the therapy is not optimal at that moment. Once treatment is successful, patients will be followed-up annually for a PSG and a device check.

Based on this paper, it can be concluded that UAS therapy is a promising novel and evolving non-CPAP treatment option for patients diagnosed with OSA. Patient screening and selection is of utmost importance in order to increase treatment outcome. The introduction of a clinical pathway including a proper patient selection can optimize both the efficacy and the workflow of this innovative therapy for selected patients with moderate to severe OSA.

## Author contributions

OV and PV conceived the concept of the manuscript and both contributed to the manuscript. JB, MD, and SO drafted the manuscript and made contricutions in the manuscript. MW, WD, and JV contributed to the quality of the manuscript. All authors read and approved the final manuscript.

### Conflict of interest statement

OV has the following potential conflicts of interest related to the topic of the manuscript: Research support and lecture fees from Inspire Medical Systems, research grant from and consultancy for Philips Respironics, research grant and lecture fees from Somnomed, consultancy for nyxoah, research support from ReVent, research support from Nightbalance. The other authors declare that the research was conducted in the absence of any commercial or financial relationships that could be construed as a potential conflict of interest. The reviewer PD and handling Editor declared their shared affiliation.

## Abbreviations

AHI: Apnea/hypopnea index
CPAP: Continuous Positive Airway Pressure
DISE: Drug Induced Sedation Endoscopy
ENT, Ear, Nose: Throat
ESS: Epworth Sleepiness Scale
FDA: Food and Drug Administration
FOSQ: Functional Outcome Sleep Questionnaire
IPG: Implantable Pusle Generator
nXII: Hypoglossal nerve
OA: Oral Appliance
ODI: Oxygen Desaturation Index
OSA: Obstructive Sleep Apnea
PSG: Polysomnography
SpO_2_: Oxygen Saturation
STAR: Stimulation Therapy for Apnea Reduction
UA: Upper Airway
UAS: Upper Airway Stimulation
